# Supplementary material for: Technologies for the Instrumental Evaluation of Physical Function in Persons Affected by Chronic Obstructive Pulmonary Disease: A Systematic Review
Source: Sensors (Basel). 2022 Sep 1;22(17):6620. doi: 10.3390/s22176620 (PMC9459845; doi:10.3390/s22176620)
Supplement: Supplementary file 1 [file sensors-22-06620-s001.zip › COPD_technology_Supplementary Material_Table S1.pdf]

Supplementary material

Systematic Review

Technologies for the instrumental evaluation of physical function in persons affected by Chronic Obstructive Pulmonary Disease: A Systematic Review of the literature

Alberto Zucchelli [1,2,†], Simone Pancera [3,*,†], Luca Nicola Cesare Bianchi [3], Alessandra Marengoni [2,4] and Nicola Francesco Lopomo [1,*]

1 Department of Information Engineering, Università degli Studi di Brescia, Bre-scia 25123, Italy; a.zucchelli001@unibs.it
2 Aging Research Center, Department of Neurobiology, Care Sciences and Society, Karolinska Institutet, Solna SE-171 65, Sweden; alessandra.marengoni@unibs.itki.se
3 IRCCS Fondazione Don Carlo Gnocchi, Milan 20148, Italy; lubianchi@dongnocchi.it
4 Department of Clinical and Experimental Sciences, Università degli Studi di Brescia, Brescia 25123, Italy
* Correspondence: s.pancera002@unibs.it (S.P.) or nicola.lopomo@unibs.it (N.F.L.); Tel.: +39-030-29881
† These authors contributed equally to this work.

Table S1. *Search strategy for PubMed database*

1. #**1 Construct search**
("Walking"[Mesh] OR "Walking Speed"[Mesh] OR "Gait"[Mesh] OR "Motion"[Mesh] OR "Posture"[Mesh] OR "Sensitivity and Specificity"[Mesh] OR "Movement"[Mesh] OR "Lower Extremity"[Mesh] OR "Upper Extremity"[Mesh] OR "Muscle Contraction"[Mesh] OR "Muscle, Skeletal"[Mesh] OR "Muscle Strength"[Mesh] OR "Muscle Fatigue"[Mesh] OR "Exercise Tolerance"[Mesh] OR "Physical Endurance"[Mesh] OR "Time Factors"[Mesh] OR "Biomechanical Phenomena"[Mesh] OR "Acceleration"[Mesh] OR "Energy Metabolism"[Mesh] OR "Motor Activity"[Mesh] OR "Physical Functional Performance"[Mesh] OR "Joints"[Mesh] OR "role function"[tiab] OR "activit*"[tiab] OR "physical"[tiab] OR "function*"[tiab] OR "performance"[tiab] OR "biomechanic*"[tiab] OR "functional screening"[tiab] OR "implementation"[tiab] OR "step*"[tiab] OR "stand*"[tiab] OR "walk*"[tiab] OR "distance"[tiab] OR "balance"[tiab] OR "grip"[tiab] OR "handgrip"[tiab] OR "sit"[tiab] OR "lift*"[tiab] OR "gait"[tiab] OR "locomot*"[tiab] OR "stair*"[tiab] OR "rise"[tiab] OR "elevation"[tiab] OR "joint kine*"[tiab] OR "sit to stand"[tiab] OR "velocity"[tiab] OR "speed"[tiab] OR "power"[tiab] OR "rate of force development"[tiab] OR "strength evaluation"[tiab] OR "strength assessment"[tiab] OR "muscle evaluation"[tiab] OR "muscle assessment"[tiab] OR "endurance evaluation"[tiab] OR "endurance assessment"[tiab] OR "normative data"[tiab] OR "kinematic characteristic*"[tiab] OR "measurement propert*"[tiab] OR "clinical evaluation"[tiab] OR "clinical assessment"[tiab] OR "motion"[tiab] OR "motor"[tiab])
2. #**2 Population search**
("pulmonary disease, chronic obstructive"[Mesh] OR "Pulmonary emphysema"[Mesh] OR "chronic obstructive pulmonary disease*"[Title/Abstract] OR "chronic obstructive lung

disease*"[Title/Abstract] OR "chronic obstructive airway disease*"[Title/Abstract] OR COPD[Title/Abstract] OR COAD[Title/Abstract])
3. #**3 Instrument search**
("Motion/instrumentation"[Mesh] OR "Actigraphy/instrumentation"[Mesh] OR "Movement/instrumentation"[Mesh] OR "Accelerometry/instrumentation"[Mesh] OR "Accelerometry/methods"[Mesh] OR "Software"[Mesh] OR "Mobile Applications"[Mesh] OR "Cell Phone"[Mesh] OR "Computers, Handheld"[Mesh] OR "Walk Test/instrumentation"[Mesh] OR "Micro-Electrical-Mechanical Systems"[Mesh] OR "Wearable Electronic Devices"[Mesh] OR "Technology/instrumentation"[Mesh] OR "Biomedical Technology"[Mesh] OR "Gait Analysis"[Mesh] OR "Exercise Test/instrumentation"[Mesh] OR "Exercise Test/methods"[Mesh] OR "Exercise Therapy/instrumentation"[Mesh] OR "Muscle Contraction/instrumentation"[Mesh] OR "Muscle Strength Dynamometer"[Mesh] OR "Resistance Training/instrumentation"[Mesh] OR "Equipment and Supplies/rehabilitation"[Mesh] OR "Equipment and Supplies/instrumentation"[Mesh] OR "Equipment and Supplies/methods"[Mesh] OR "Clinical Nursing Research/instrumentation"[Mesh] OR "Spectroscopy, Near-Infrared"[Mesh] OR "Electromyography/instrumentation"[Mesh] OR "near infrared spectroscopy"[tiab] OR "NIRS"[tiab] OR "gait analysis"[tiab] OR "performance-based test"[tiab] OR "wearable*"[tiab] OR "pedometer"[tiab] OR "acceleromet*"[tiab] OR "instrument*"[tiab] OR "tool*"[tiab] OR "smartphone*"[tiab] OR "smart-phone*"[tiab] OR "technolog*" OR "kinematic analysis"[tiab] OR "kinetic analysis"[tiab] OR "kinematic parameters"[tiab] OR "robot*"[tiab] OR "virtual"[tiab] OR "motion analysis"[tiab] OR "inertial sensor*"[tiab] OR "quantitative measure*"[tiab] OR "quantitative analysis"[tiab] OR "electromyograph*"[tiab] OR "EMG"[tiab])
4. #**4 Filter for measurement properties**
(instrumentation[sh] OR methods[sh] OR Validation Study[pt] OR Comparative Study[pt] OR ″psychometrics″[Mesh] OR psychometr*[tiab] OR clinimetr*[tw] OR clinometr*[tw] OR "outcome assessment, health care"[Mesh] OR outcome assessment[tiab] OR outcome measure*[tw] OR ″observer variation″[Mesh] OR observer variation[tiab] OR ″Health Status Indicators″[Mesh] OR ″reproducibility of results″[Mesh] OR reproducib*[tiab] OR ″discriminant analysis″[Mesh] OR reliab*[tiab] OR unreliab*[tiab] OR valid*[tiab] OR coefficient[tiab] OR homogeneity[tiab] OR homogeneous[tiab] OR ″internal consistency″[tiab] OR (cronbach*[tiab] AND (alpha[tiab] OR alphas[tiab])) OR (item[tiab] AND (correlation*[tiab] OR selection*[tiab] OR reduction*[tiab])) OR agreement[tiab] OR precision[tiab] OR imprecision[tiab] OR ″precise values″[tiab] OR test–retest[tiab] OR (test[tiab] AND retest[tiab]) OR (reliab*[tiab] AND (test[tiab] OR retest[tiab])) OR stability[tiab] OR interrater[tiab] OR inter-rater[tiab] OR intrarater[tiab] OR intra-rater[tiab] OR intertester[tiab] OR inter-tester[tiab] OR intratester[tiab] OR intra-tester[tiab] OR interobserver[tiab] OR inter-observer[tiab] OR intraobserver[tiab] OR intraobserver[tiab] OR intertechnician[tiab] OR inter-technician[tiab] OR intratechnician[tiab] OR interexaminer[tiab] OR inter-examiner[tiab] OR intraexaminer[tiab] OR intra-examiner[tiab] OR interassay[tiab] OR inter-assay[tiab] OR intraassay[tiab] OR intra-assay[tiab] OR interindividual[tiab] OR inter-individual[tiab] OR intraindividual[tiab] OR intra-individual[tiab] OR interparticipant[tiab] OR inter-participant[tiab] OR intraparticipant[tiab] OR intra-participant[tiab] OR kappa[tiab] OR kappa′s[tiab] OR kappas[tiab] OR repeatab*[tiab] OR ((replicab*[tiab] OR repeated[tiab]) AND (measure[tiab] OR measures[tiab] OR findings[tiab] OR result[tiab] OR results[tiab] OR test[tiab] OR tests[tiab])) OR generaliza*[tiab] OR generalisa*[tiab] OR concordance[tiab] OR (intraclass[tiab]

AND correlation*[tiab]) OR discriminative[tiab] OR ‘‘known group’’[tiab] OR factor analysis[tiab] OR factor analyses[tiab] OR dimension*[tiab] OR subscale*[tiab] OR (multitrait[tiab] AND scaling[tiab] AND (analysis[tiab] OR analyses[tiab])) OR item discriminant[tiab] OR interscale correlation*[tiab] OR error[tiab] OR errors[tiab] OR ‘‘individual variability’’[tiab] OR (variability[tiab] AND (analysis[tiab] OR values[tiab])) OR (uncertainty[tiab] AND (measurement[tiab] OR measuring[tiab])) OR ‘‘standard error of measurement’’[tiab] OR sensitiv*[tiab] OR responsive*[tiab] OR ((minimal[tiab] OR minimally[tiab] OR clinical[tiab] OR clinically[tiab]) AND (important[tiab] OR significant[tiab] OR detectable[tiab]) AND (change[tiab] OR difference[tiab])) OR (small*[tiab] AND (real[tiab] OR detectable[tiab]) AND (change[tiab] OR difference[tiab])) OR meaningful change[tiab] OR ‘‘ceiling effect’’[tiab] OR ‘‘floor effect’’[tiab] OR ‘‘Item response model’’[tiab] OR IRT[tiab] OR Rasch[tiab] OR ‘‘Differential item functioning’’[tiab] OR DIF[tiab] OR ‘‘computer adaptive testing’’[tiab] OR ‘‘item bank’’[tiab] OR ‘‘cross-cultural equivalence’’[tiab])
5. #**5 Exclusion filter**
(‘‘biography’’[Publication Type] OR ‘‘case reports’’[Publication Type] OR ‘‘comment’’[Publication Type] OR ‘‘directory’’[Publication Type] OR ‘‘editorial’’[Publication Type] OR ‘‘festschrift’’[Publication Type] OR ‘‘interview’’[Publication Type] OR ‘‘legislation’’[Publication Type] OR ‘‘letter’’[Publication Type] OR ‘‘news’’[Publication Type] OR ‘‘newspaper article’’[Publication Type] OR ‘‘patient education handout’’[Publication Type] OR ‘‘consensus development conference’’[Publication Type] OR ‘‘consensus development conference, nih’’[Publication Type] OR ‘‘practice guideline’’[Publication Type]) NOT (‘‘animals’’[Mesh Terms] NOT ‘‘humans’’[Mesh Terms])
6. Combination: #**1 AND** #**2 AND** #**3 AND** #**4 NOT** #**5**
